# Subthalamic Stimulation Improves Short-Term Satisfaction with Life and Treatment in Parkinson’s Disease

**DOI:** 10.3390/jpm14101023

**Published:** 2024-09-26

**Authors:** Anna Sauerbier, Pia Bachon, Leire Ambrosio, Philipp A. Loehrer, Alexandra Rizos, Stefanie T. Jost, Alexandra Gronostay, Gereon R. Fink, Keyoumars Ashkan, Christopher Nimsky, Veerle Visser-Vandewalle, K. Ray Chaudhuri, Lars Timmermann, Pablo Martinez-Martin, Haidar S. Dafsari

**Affiliations:** 1 University of Cologne, Faculty of Medicine and University Hospital Cologne, Department of Neurology, 50937 Cologne, Germany; pbachon@smail.uni-koeln.de (P.B.); a.rizos@nhs.net (A.R.); stefanie.jost@uk-koeln.de (S.T.J.); alexandra.gronostay@klinikum-lev.de (A.G.); neurologie-direktor@uk-koeln.de (G.R.F.); 2 Institute of Psychiatry, Psychology and Neuroscience, King’s College London, London SE5 8AF, UK; ray.chaudhuri@nhs.net; 3 NIHR Applied Research Collaboration Wessex, School of Health Sciences, University of Southampton, Southampton SO17 1BJ, UK; l.ambrosio-gutierrez@soton.ac.uk; 4 Department of Neurology, University Hospital Giessen and Marburg, Campus Marburg, 35043 Marburg, Germany; philipp.loehrer@uk-gm.de (P.A.L.); lars.timmermann@uk-gm.de (L.T.); 5 Parkinson Foundation International Centre of Excellence, King’s College Hospital, London SE5 8AF, UK; k.ashkan@nhs.net; 6 Cognitive Neuroscience, Institute of Neuroscience and Medicine (INM-3), Research Centre Jülich, 52428 Jülich, Germany; 7 Department of Neurosurgery, University Marburg, 35043 Marburg, Germany; christopher.nimsky@uk-gm.de; 8 University of Cologne, Faculty of Medicine and University Hospital Cologne, Department of Stereotactic and Functional Neurosurgery, 50937 Cologne, Germany; veerle.visser-vandewalle@uk-koeln.de; 9 Center for Networked Biomedical Research in Neurodegenerative Diseases (CIBERNED), Carlos III Institute of Health, 28029 Madrid, Spain; pmm650@hotmail.com

**Keywords:** treatment satisfaction, life satisfaction, quality of life, deep brain stimulation, non-motor symptoms

## Abstract

The effect of subthalamic stimulation (STN-DBS) on patients’ personal satisfaction with life and their Parkinson’s disease (PD) treatment is understudied, as is its correlation with quality of life (QoL). Therefore, we tested the hypothesis that STN-DBS for PD enhances satisfaction with life and treatment. In a prospective, multicenter study with a 6-month follow-up involving 121 patients, we measured the main outcomes using the Satisfaction with Life and Treatment Scale (SLTS-7). Secondary outcomes included the eight-item PD Questionnaire (PDQ-8), European QoL Questionnaire (EQ-5D-3L), EQ-Visual Analogue Scale (VAS), Non-Motor Symptom Scale (NMSS), Hospital Anxiety and Depression Scale (HADS), and Unified PD Rating Scale (UPDRS). Longitudinal outcome changes, effect sizes (Cohen’s d), and correlations between outcome changes were analyzed. SLTS-7 scores improved at the 6-month follow-up, particularly in the domains of ‘satisfaction with physical health’ and ‘satisfaction with treatment’. Change scores correlated strongly (EQ-VAS), moderately (PDQ-8 SI and HADS), and weakly (UPDRS-activities of daily living and EQ-5D-3L) with other scales. Satisfaction with physical health, psychosocial well-being, or treatment was not related to UPDRS-motor examination. This study provides evidence that STN-DBS enhances patients’ personal satisfaction with life and treatment. This satisfaction is associated with improvements in the QoL, daily activities, and neuropsychiatric aspects of PD rather than its motor aspects.

## 1. Introduction

Subthalamic nucleus deep brain stimulation (STN-DBS) is a safe and effective treatment in patients with medication-refractory tremor or motor complications in advanced stages of Parkinson’s disease (PD), improving quality of life (QoL), non-motor, and motor symptoms [1,2]. Studies on the clinical efficacy of DBS mainly focus on QoL using the disease-specific Parkinson’s Disease Questionnaire (PDQ) or the more generic European Quality of Life Questionnaire with 5 dimensions (EQ-5D) [3,4]. However, these tools do not consider the concept of “life satisfaction”. While QoL has been reported to be mainly influenced by external circumstances, life satisfaction depends on subjective factors reflecting “how much the person likes the life he/she leads” [5,6,7,8] and covers cognitive aspects of well-being of patients based on subjective factors. A better understanding of satisfaction with life and treatment will identify aspects that are personally meaningful to the patient and therefore will improve their overall well-being and quality of life.

To date, there is a scarcity of studies on life satisfaction in PD, in particular there is a lack of studies using scales validated in patients with PD [9]. Therefore, one of the main emphases of this manuscript is the effect of DBS on satisfaction with life and treatment besides the effect on quality of life.

Our group has recently validated the Satisfaction with Life and Treatment Scale-7 (SLTS-7), which allows a PD specific assessment of physical, psycho-social, and treatment satisfaction [10]. The objectives of this study were: (1) to investigate the effect of STN-DBS on satisfaction with life and treatment 6-months following STN-DBS and (2) to assess association of changes in life and treatment satisfaction with improvements of QoL, motor, and non-motor aspects at a 6-month follow-up after STN-DBS. 

## 2. Materials and Methods

### 2.1. Study Design and Ethical Approval

This study is part of an ongoing, prospective, observational, multicenter study including patients undergoing DBS for PD (Non-motor International Longitudinal Study, NILS) [11,12]. Before study inclusion all patients gave written informed consent. The study was performed following the Declaration of Helsinki and the protocols were approved by local ethics committees (Cologne, 12/145; Marburg: Study no. 155/17).

### 2.2. Participants

PD was diagnosed according to the UK Brain Bank criteria [13]. Screening and indication evaluations for advanced treatments in the DBS group were conducted according to international guidelines as described in former publications by our group [10].

### 2.3. Clinical Assessment

Patients were assessed preoperatively in the medication on-state (MedON) and postoperatively at 6-month follow-up in the medication and stimulation on-state (MedON/StimON). Assessments were conducted at thirty minutes after levodopa intake [14].

The following scale was conducted as main outcome parameter:

Satisfaction with Life and Treatment Scale-7

The SLTS-7 is a recently validated, modified version on the patient-completed SLS-6 [10,15]. SLTS-7 includes seven items assessing satisfaction including the following: life as a whole (item 1), physical health (item 2), psychological well-being (item 3), social relations (item 4), leisure (item 5), and additionally, Parkinson treatment (item 6). In items 1–6 the wording is “All things considered, how satisfied are you with…?”. Another question surveys the expectations met in relation to treatment (item 7). In Item 7 the wording is “All things considered: Does the treatment so far meet your expectations?“ Item scores range from 1 (not at all) to 10 (totally). 

In our recently published validation study, we performed an exploratory factor analysis which produced item 2 (namely physical), items 3–5 (psycho-social) and items 6–7 (treatment) as three independent domains [10]. A composite score representing the satisfaction level from these three domains (SLTS-7 composite score) was calculated as the sum of their respective scores. Therefore, the SLTS-7 allows the evaluation of the following: a direct overall score (item 1 only; range 1–10), scores for three specific domains (physical, psycho-social, and treatment satisfaction, respectively ranging from 1–10, 3–30, and 2–20), and a composite score summarizing these three domains (SLTS-7 composite score ranging from 6–60). 

The following tools were included as secondary outcomes:

Quality of life

The 8-item Parkinson’s Disease Questionnaire (PDQ-8) is an abridged version of the PDQ-39 for self-evaluation of eight dimensions which contribute to QoL. The scale has been commonly used in advanced PD cohorts undergoing DBS and is also recommended by the International Parkinson and Movement Disorder Society [4,16,17]. The scale captures mobility, activities of daily living, emotional well-being, social support, cognition, communication, bodily discomfort, and stigma. The PDQ-8 is a commonly used scale in DBS studies [12,18,19]. The results are expressed as summary index (SI) which ranges between 0 (no impairment) and 100 (maximum impairment) [3].

The EQ-5D-3L with 5 dimensions was developed by the EuroQol Group. It is a generic measure that assesses 5 aspects of QoL: mobility, self-care, daily activities, pain and discomfort, and anxiety and depression. Each item is rated on a three-level Likert-scale resulting in a five-digit Health State from “11111“ (completely healthy) to “33333“ (seriously ill) which can be converted into a SI from 0 (death) to 1 (best health state). Further, negative values for health states are possible, which are considered worse than death. This conversion was performed using a country-specific value set for Germany and expressed as EQ-5D Time-Trade-Off (TTO) [20].

The EQ-VAS is a visual analogue scale where patients indicate their current health-related QoL from 0 (worst imaginable health state) to 100 (best imaginable health state) [20].

Non-motor aspects

The 30-item rater-based Non-Motor Symptom Scale (NMSS) is divided into nine domains of PD symptoms: (1) cardiovascular, (2) sleep/fatigue, (3) mood/apathy, (4) perceptual problems/hallucinations, (5) attention/memory, (6) gastrointestinal tract, (7) urinary, (8) sexual function, and (9) miscellaneous (including pain, inability to smell/taste, weight changes, and sweating). This assessment refers to the non-motor symptoms of the last 4 weeks. The NMSS is a commonly used scale in DBS studies [21,22,23]. The NMSS total score ranges from 0 (no non-motor symptoms) to 360 (maximum impairment due to non-motor symptoms). The total score can be used to classify the severity of the NMSs’ total burden (0 = none, 1–20 = mild, 21–40 = moderate, 41–70 = severe, and ≥70 = very severe) [24,25].

Motor impairment

The Unified Parkinson’s Disease Rating Scale (UPDRS 3.0) Parts I to IV was used to assess mental dysfunctions, activities of daily living, motor examination, and motor complications, respectively. UPDRS-I (cognition, behavior, and mood), -II (activities of daily living), -III (motor examination), and –IV (motor complications) scores range from 0 (no impairment) to respectively 16, 48, 108, and 23 (maximum impairment) [26].

Medication requirements

The levodopa equivalent daily dose (LEDD) was used to assess medication requirements according to the Jost method [27].

### 2.4. Statistical Analysis

We tested for normal distribution of each variable using the Shapiro–Wilk test. To analyze longitudinal changes, we applied Wilcoxon signed-rank test or Student’s paired *t*-test, when criteria for parametric tests were fulfilled. 

Additionally, we calculated change scores (Test_baseline_ − Test_6-month follow-up_) of all clinical parameters and then computed Spearman correlations between change scores of the SLTS-7 and other outcomes. The strength of the correlations were defined as follows: ‘negligible’ r_s_ ≤ 0.19, ‘weak’ r_s_ = 0.20–0.39, ‘moderate’ r_s_ = 0.40–0.59, ‘strong’ r_s_ = 0.60–0.79, and ‘very strong’ r_s_ = 0.80–1.00 [28].

Furthermore, to investigate the strength of clinical responses, we calculated the relative changes (mean test_baseline_ − mean test_follow-up_/mean test_baseline_ × 100) and Cohens’s *d* effect size (|mean test_baseline_ − mean test_follow-up_|/SD_pooled_) [29]. An effect size (*d*) ≥ 0.80 is considered a “large effect”, 0.50–0.79 a “moderate effect”, and 0.20–0.49 a “small effect” [30].

All analyses were performed using Statistical Package for Social Science (SPSS Version 27.0 for Windows) and *p* values < 0.05 were rated statistically significant.

## 3. Results

**Figure 1:**
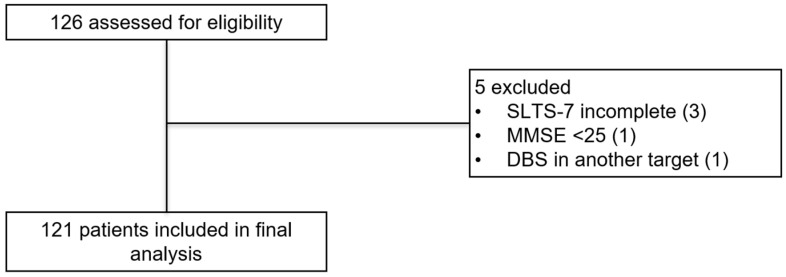
Recruitment process.

121 patients (62.8% male) undergoing bilateral STN-DBS for PD were included in this study (see Figure 1). The mean age at baseline was 62.2 ± 8.3 years and the mean disease duration was 9.8 ± 4.7 years.

### 3.1. Changes of Outcome Parameters from Baseline to 6-Month Follow-Up

**Figure 2:**
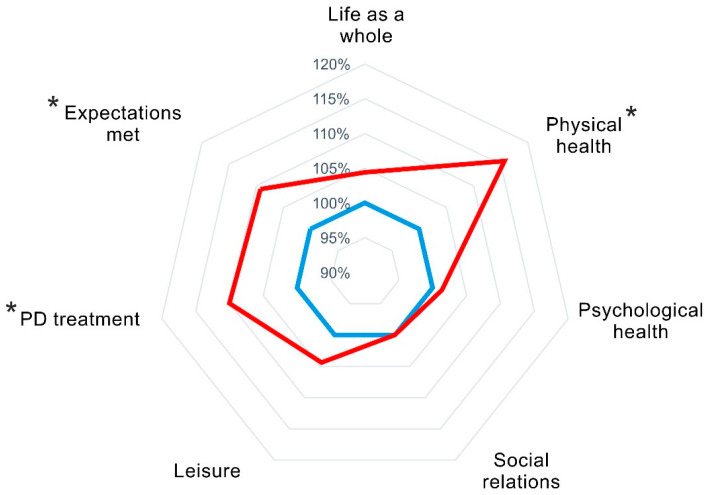
Satisfaction with Life and Treatment Scale-7 (SLTS-7) at preoperative baseline and 6-month follow-up. Abbreviations: PD = Parkinson’s Disease and SLTS-7 = Satisfaction with Life and Treatment Scale. SLTS-7 item scores are normalized to baseline scores per subject. Blue: baseline, red: 6-month follow-up. The larger area of the red relative to the blue reflects an SLTS-7 item improvement. *: significant changes from baseline to 6-month follow-up.

Clinical longitudinal changes are presented in Table 1. The SLTS-7 composite score improved at 6-month follow-up. Post-hoc analyses of SLTS-7 items showed improvements of the satisfaction with physical health and Parkinson treatment and indicated that the expectations regarding treatment of PD were met (see Figure 2). Relative changes and effect sizes are shown in Table 2. The relative change was highest for satisfaction with physical health with an improvement of 15.7%, followed by an improvement of 10% for satisfaction with PD treatment and 9.2% for expectations met. The effect sizes were ‘small’ for the SLTS-7 composite score and SLTS-7 physical health and treatment domains.

Furthermore, all secondary outcome parameters improved at 6-month follow-up. 

### 3.2. Correlation Analyses

Table 3 shows the results of Spearman correlations between the change scores of the SLTS-7 composite score and all other outcome parameters. Regarding QoL outcomes, these correlations were ‘strong’ for the EQ-VAS, ‘moderate’ for the PDQ-8 SI, and ‘weak’ for the EQ-5D-3L TTO. Regarding the motor aspects of PD, the correlations were ‘weak’ with the UPDRS-II and not significant for the UPDRS-III and -IV. Regarding the non-motor aspects of PD, these correlations were ‘moderate’ for the HADS total score and ‘weak’ for the NMSS total score. 

Furthermore, explorative post-hoc correlation analyses for the HADS domains and SLTS-7 composite score changes were ‘moderate’ for HADS-depression (r_s_ = −0.48, *p* < 0.001) and ‘weak’ for HADS-anxiety (r_s_ = −0.34, *p* < 0.001). Explorative correlations between changes of the SLTS-7 composite score and NMSS domains were ‘weak’ for the sleep domain (r_s_ = −0.24, *p* = 0.008) and the mood/apathy domain (r_s_ = −0.29, *p* = 0.002). 

Table 3 also shows correlation analyses between the changes in SLTS-7 domains and all other clinical outcome parameters. ‘Moderate’ correlations were observed for the following: (1) changes in satisfaction with physical health and psycho-social aspects with EQ-VAS and (2) treatment satisfaction with PDQ-8 SI. This section may be divided by subheadings. It should provide a concise and precise description of the experimental results, their interpretation, as well as the experimental conclusions that can be drawn.

## 4. Discussion

In this prospective, observational, multicenter study including 121 patients undergoing DBS for PD with a 6-month follow-up, we observed postoperative improvement in satisfaction with life and treatment, which was associated with improvements of quality of life (PDQ-8 SI), subjective overall health status (VAS), anxious and depressive mood (HADS), and activities of daily living (UPDRS-II).

### 4.1. Overall Satisfaction with Life and Treatment

We observed an improvement of overall satisfaction with life and treatment (SLTS-7 composite score) after STN-DBS. Post-hoc tests showed that this improvement was driven by improvements of the “physical health”, “Parkinson’s disease treatment”, and “Expectations met” domains, which improved postoperatively between 9.2% and 15.7% (small effect sizes). 

### 4.2. Satisfaction with Physical Health

Satisfaction with physical health improved at 6-month follow-up. This is in line with previous studies reporting a postoperative improvement in satisfaction with motor function at short-term follow-up [9]. 

### 4.3. Satisfaction with Parkinson Treatment and Expectations Met

As expected, “treatment satisfaction” and “treatment expectations met” improved postoperatively at 6-month follow-up. This is in line with previous studies in the literature [31]. Of note, the present study extends previous reports with the observation that treatment satisfaction outcome correlated moderately with quality of life and weakly with depression and anxiety changes. 

We observed no significant association between changes of treatment satisfaction and motor symptoms or motor complications. This result confirms findings by Maier et al., who reported that improvement in motor symptoms does not result in an improved subjective global outcome [32]. In addition, a study on patient selected goals and satisfaction after STN-DBS by Nam et al. found that an improvement in motor symptoms using the UPDRS-III was not significantly associated with patient overall treatment satisfaction when applying the patient global impression of improvement [33]. 

### 4.4. Satisfaction with Psychological Health 

Confirming results by Ferrera at al. [9], we observed no improvement of satisfaction with psychological health in patients undergoing DBS for PD. However, depression and anxiety improved postoperatively, which correlated with an improvement of the QoL. A possible explanation may be that depression and anxiety did not improve to a degree that was subjectively satisfactory. Another possible reason may be that other psychological aspects of PD, such as impulsivity, apathy, or alexithymia, which were not investigated in this study, contributed to the satisfaction with psychological health of patients [11]. Further studies are needed to identify the reasons for this observation.

### 4.5. Satisfaction with Social Relations, Leisure, and Life as a Whole

Confirming results of a small pilot study (*n* = 21) by Ferrara et al. [9], we observed no improvement of aspects of social relations, leisure, and life as a whole. Schüpbach et al. reported an increase in difficulties with social interactions in patients who underwent STN-DBS, including their spouses, their families, and their socio-professional environment [34]. As a possible explanation, the authors discussed the sudden and profound changes in their existence following successful neurosurgery. Following this argument, a review by Bell et al. summarized that patients may experience problems with self-identification following DBS given that motor symptoms, an important aspect dictating their life and their interactions with other people, suddenly and drastically change [35]. A successful postoperative adjustment to existential changes may contribute to satisfaction with social relations, leisure, and life as a whole.

### 4.6. Relationship of Life and Treatment Satisfaction with Other Clinical Parameters

We explored the relationship between the changes of the SLTS-7 composite score and other clinical outcome parameters. Underlining the close connection between a person’s life satisfaction and subjective well-being, we observed a strong association between improvements of the SLTS-7 and the EQ-VAS [36]. Furthermore, improvements of satisfaction with life and treatment (SLTS-7) and quality of life (PDQ-8 SI) were moderately correlated, which is in line with previous studies on the differences of the theoretical constructs of these two parameters: quality of life is mainly driven by external factors, whereas life satisfaction focusses on internal and subjective characteristics [7,37]. This difference has also been outlined in our recent SLTS-7 validation study, which showed that constructs of life and treatment satisfaction and quality of life differed [10]. In particular, we found that life and treatment satisfaction (SLTS-7) was more closely associated with total motor symptoms’ severity and the quality of life (PDQ-8 SI) with total non-motor symptoms burden.

In contrast to our present observation that in patients undergoing DBS for PD, improvements of life satisfaction and QoL are related, a pioneer study by Hasegawa et al. reported no correlation between these parameters, possibly due to the small cohort size (*n* = 22) or the use of a satisfaction questionnaire for which the psychometric properties were not validated [31]. Furthermore, we found a moderate correlation between the improvements in life and treatment satisfaction and activities of daily living. This might be explained by the fact that improved activities of daily living are linked to patients’ independence and self-efficacy, which in turn is closely related to higher life satisfaction [38,39]. Of note, we did not find a significant association between an improvement in motor symptoms and life and treatment satisfaction. This underlines that an objective improvement in motor symptoms may not suffice for a subjectively measured improved life and treatment satisfaction [32,33]. It appears that motor improvement is necessary for an improvement of activities of daily living, which in turn may suffice for improved satisfaction with life and treatment. This was also found by Ferrara et al., who found health-related quality of life benefits measured with the Questions on Life Satisfaction modular questionnaire (QLS^M^) correlated with improvement in activities of daily living but not pure motor symptoms [9]. 

Furthermore, in line with previous studies in patients undergoing DBS, we found that depression and anxiety (NMSS mood domain and HADS) play an important role in the improvement of satisfaction with life and treatment [9]. This confirms results by Rosquist et al. who reported that worse depression is associated with poorer life satisfaction in a PD population [40], and findings by Jonasson et al., who reported that worse depression predicts worse life satisfaction in a general PD population [39]. Furthermore, we also found a relationship between the NMSS sleep domain and SLTS-7 outcomes. This is in line with previous studies, which found that treatment satisfaction is hampered in patients with insomnia [41].

### 4.7. Limitations

Several limitations of this study have to be discussed. Firstly, we have to acknowledge a relatively small sample size (*n* = 121), even though within the currently available literature this is the biggest study available on satisfaction with life and treatment in a PD population undergoing DBS. Secondly, this was not a randomized controlled study and the results from our study only address short-term effects. Therefore, studies using the SLTS-7 looking at long-term effects, e.g., over a 5-year period, in a randomized controlled design, are needed. The effect size of STN-DBS on the SLTS-7 is small in our sample. We assessed the effect size based on Cohen’s *d* [42] because a minimal clinically important difference (MCID) for the SLTS-7 has not yet been established. Further studies are needed to identify its MCID. Specific parameters, such as social support, which might influence life and treatment satisfaction, were not investigated in our study [38]. Further studies are needed on gender differences in clinically efficacy of DBS on satisfaction with life and treatment and the relationship to gender differences in referrals for DBS treatment [29,43]. Moreover, as mentioned above, specific neuropsychiatric aspects of PD, such as impulsivity, apathy, and alexithymia were not systematically assessed in this study, but may impact satisfaction with psychological health in patients undergoing STN-DBS for PD. Furthermore, in our study we have only included patients who underwent STN-DBS. As the effects of DBS on QoL differ between STN and other surgical target regions, such as the globus pallidus internus [44], differential effects may also be observed for satisfaction with life and treatment outcome. Further studies comparing the effects in various surgical target regions are needed.

## 5. Conclusions

This prospective, multicenter study provides evidence that overall satisfaction with life and treatment, in particular satisfaction with physical health and treatment satisfaction, improve at 6-month follow-up in patients undergoing STN-DBS for PD. The improvement in life and treatment satisfaction is associated with an improvement in subjective health status, quality of life, activities of daily living, and non-motor symptoms, but not with improvements of motor impairment. These results highlight the importance of holistic assessments of patients undergoing DBS, including both quality of life and satisfaction with life and treatment. In the future, the value of a “life satisfaction” assessment using the SLTS-7 is the additional identification of factors which contribute to patients’ subjectively perceived well-being in patients undergoing DBS for PD.

## Figures and Tables

**Table 1:** Baseline and 6-month follow-up characteristics.

	Baseline			6-Month Follow-Up			
	N	Mean	SD	N	Mean	SD	*p*
SLTS-7 composite (Items 2–7)	121	39.5	10.2	121	41.9	12.0	**0.04**
Satisfaction with							
1. Life as a whole	121	6.8	2.0	121	7.1	1.9	0.100
2. Physical health	121	5.1	2.2	121	5.9	2.0	**<0.001**
3. Psychological health	121	6.9	2.2	121	7.0	2.3	0.624
4. Social relations	121	7.2	2.2	121	7.2	2.2	0.675
5. Leisure	121	6.8	2.2	121	7.1	2.1	0.168
6. PD treatment	121	7.0	2.2	121	7.7	2.0	**0.003**
7. Expectations met	121	6.5	2.2	121	7.1	2.5	**0.035**
Psycho-social well-being domain (Items 3–5)	121	20.9	5.7	121	21.3	6.1	0.224
Treatment domain (Items 6–7)	121	13.6	4.2	121	14.8	4.2	**0.007**
PDQ-8 Summary Index	121	31.9	15.7	120	24.4	16.1	**<0.001**
EQ-5D-3L TTO *	119	0.8	0.2	120	0.8	0.2	**0.001**
EQ-VAS	118	57.2	19.6	119	64.4	19.1	**0.001**
NMSS total	121	56.3	31.1	120	39.1	25.2	**<0.001**
HADS total	120	10.5	6.3	121	8.9	6.2	**0.003**
UPDRS total	120	24.0	10.7	113	18.0	9.2	**<0.001**
Part I: cognition, behavior, mood	121	1.9	1.7	121	1.6	1.3	0.281
Part II: activities of daily living	121	12.5	5.9	121	9.7	5.4	**<0.001**
Part III: motor examination	120	24.0	10.7	113	18.0	9.2	**<0.001**
Part IV: motor complications	121	6.9	3.5	121	3.7	3.2	**<0.001**
LEDD total	121	1125.4	529.6	121	536.6	296.9	**<0.001**

Significant results are highlighted in bold font. Within group changes from baseline to 6-month follow-up were analyzed with Wilcoxon signed-rank tests. Post-hoc, we explored the SLTS-7 items and composite scores. Abbreviations: EQ-5D-3L = European Quality of Life Questionnaire with 5 Dimensions and 3 Levels; HADS = Hospital Anxiety and Depression Scale; LEDD = Levodopa Equivalent Daily Dose; NMSS = Non-Motor Symptom Scale; N = Number; PDQ-8 = eight-item Parkinson’s Disease Questionnaire; SD = Standard Deviation; SLTS-7 = Satisfaction with Life and Treatment Scale-7; TTO = Time-Trade-Off; UPDRS = Unified Parkinson’s Disease Rating Scale; VAS = Visual Analogue Scale. * Adapted to Country according to EuroQol.

**Table 2:** Relative changes and effect sizes (Cohen’s d) from baseline to 6-month follow-up.

	Relative Change (%)	Cohen’s d	Classification of Effect Size
SLTS-7 composite (Items 2–7)	6.1	0.23	small
Satisfaction with			negligible
1. Life as a whole	4.4	0.15	negligible
2. Physical health	15.7	0.38	small
3. Psychological health	1.4	0.04	negligible
4. Social relations	0.0	0.00	negligible
5. Leisure	4.4	0.14	negligible
6. PD treatment	10.0	0.33	small
7. Expectations met	9.2	0.26	small
Psycho-social well-being domain (3–5)	1.9	0.07	negligible
Treatment domain (7–8)	8.8	0.29	small

Relative change = (mean_baseline_ − mean_6-month follow-up_)/mean test_baseline_ × 100. Cohen’s d = (mean_baseline_ − mean_6-month follow-up_)/SD_pooled_. Effect size: ‘small’ 0.20–0.49, ‘moderate’ 0.50–0.79, and ‘large’ ≥ 0.80. Abbreviation: SLTS-7 = Satisfaction with Life and Treatment Scale-7.

**Table 3:** Spearman correlations between change scores at 6-month follow-up.

	SLTS-7 Composite (Items 2–7)	Domains of SLTS-7		
		Physical Health	Psycho-Social Well-Being	Treatment
PDQ-8 Summary Index	**−0.49**	**−0.33**	**−0.42**	**−0.47**
EQ-5D-3L TTO *	**0.37**	**0.38**	**0.31**	**0.26**
EQ- VAS	**0.64**	**0.47**	**0.55**	**0.35**
NMSS total	**−0.34**	**−0.26**	**−0.24**	**−0.24**
HADS total	**−0.46**	**−0.38**	**−0.40**	**−0.38**
UPDRS total	**−0.25**	**−0.23**	**−0.19**	**−0.28**
Part I: cognition, behavior, mood	**−0.26**	−0.07	**−0.28**	**−0.20**
Part II: activities of daily living	**−0.39**	**−0.36**	**−0.27**	**0.33**
Part III: motor examination	−0.01	0.02	−0.03	−0.09
Part IV: motor complications	−0.11	−0.17	−0.03	−0.08
LEDD	0.02	−0.07	0.04	0.10

Coefficients for significant correlations are highlighted in bold font. Strength of the Spearman correlations: ‘negligible’ r_s_ ≤ 0.19, ‘weak’ r_s_ = 0.20–0.39, ‘moderate’ r_s_ = 0.40–0.59, ‘strong’ r_s_ = 0.60–0.79, and ‘very strong’ r_s_ = 0.80–1.00. Greater improvements of SLTS-7 composite were correlated to greater improvements of EQ-VAS (‘strong’), PDQ-8 SI, HADS total (all ‘moderate’) as well as EQ-5D-3L TTO, NMSS total, UPDRS total and UPDRS ‘cognition, behavior, mood’ and ‘activities of daily living’ (all ‘weak’). Abbreviations: EQ-5D-3L = European Quality of Life Questionnaire with 5 Dimensions and 3 Levels; HADS = Hospital Anxiety and Depression Scale; LEDD = Levodopa Equivalent Daily Dose; NMSS = Non-Motor Symptom Scale; PDQ-8 = eight-item Parkinson’s Disease Questionnaire; SLTS-7 = Satisfaction with Life and Treatment Scale-7; TTO = Time-Trade-Off; UPDRS = Unified Parkinson’s Disease Rating Scale; VAS = Visual Analogue Scale. * Adapted to Country according to EuroQol.

## Data Availability

The data used to support the findings of this study are available from the corresponding author upon reasonable request.
